# Editorial: Insights in ELSI in science and genetics 2024–2025

**DOI:** 10.3389/fgene.2026.1897499

**Published:** 2026-07-10

**Authors:** Dov Greenbaum

**Affiliations:** 1 Department of Molecular Biophysics and Biochemistry, Yale University, New Haven, CT, United States; 2 Zvi Meitar Institute for Legal Implications of Emerging Technologies, Harry Radzyner Law School, Reichman University, Herzliya, Israel

**Keywords:** anticipatory governance, data sovereignty, ELSI, ethical scalability, genomic governance, global bioethics, responsible research and innovation

## 1 Introduction

When the Human Genome Project set aside a portion of its budget for research on ethical, legal, and social implications, ELSI emerged as a distinctive institutional experiment: an effort to build reflection on genomics into the scientific enterprise itself. Initially a U.S.-led innovation within a heavily American but internationally collaborative scientific program, many of its early questions are now familiar: genetic testing, privacy, discrimination, informed consent, and the potential for eugenic misuse. Thirty-five years later, the field has expanded well beyond those origins, geographically, technologically, and institutionally, a broadening that has long been central to debates about the future of ELSI itself (Greenbaum, 2013; Greenbaum, 2015).

This expansion is not merely additive. It marks a shift from ELSI as a study of implications to ELSI as a practice of infrastructure‐building, a challenge, as this editorial argues, of ethical scalability as much as ethical sensitivity.

The substantive contributions to this Research Topic make that expansion visible. They span genomic medicine, artificial intelligence in healthcare, microbiome engineering, brain organoids, synthetic biology, human genetic resources, biospecimen data governance, incidental findings, variant reinterpretation, low-penetrance copy number variants, and the process of geneticization itself. Geographically, the issue brings together work focused on or authored from contexts including Japan, South Korea, China, Portugal, Greece and the Greek-speaking world, the GCC region, the United States, Canada, the Netherlands, and broader European networks.

Taken together, these contributions mark what might be called the governance turn in ELSI: a shift from evaluating technological consequences to constructing the institutional architectures through which genomic futures are shaped. This turn has parallels in broader movements toward anticipatory governance and responsible research and innovation, which similarly argue for embedding ethical and social analysis within scientific practice rather than applying it after the fact.

The collection shows a field widening in both subject matter and method. The older ELSI questions remain present, but the emphasis is increasingly on institutional design: how public and professional attitudes are measured, how responsibility is allocated across actors, how governance obligations persist over time, and how genomic policy is shaped by sovereignty, security, markets, religion, and regional legal cultures. ELSI is moving from implications to infrastructure, increasingly focusing on how to design the institutions through which society governs genomic technologies.

Mikami’s contribution to this Research Topic offers a conceptual hinge for this transition: ELSI was born within the “reading” paradigm of genomics, organized around questions of knowing, predicting, and disclosing genetic information, but the field is now entering a “writing” paradigm in which the central challenge is governing the design and synthesis of living systems (Mikami). If the Human Genome Project inaugurated the ELSI of reading genomes, contemporary biotechnology is demanding an ELSI of writing futures.

Historically, ELSI scholars have often been positioned as interpreters of consequences rather than co-designers of trajectories. The papers in this collection suggest a different emerging posture: ELSI as anticipatory, participatory, and institutionally embedded, shifting from external reflection on the consequences of genomic knowledge to collaborative participation in the choices through which genomic futures are designed.

That shift frames this editorial.

The contributions of this Research Topic cluster around three important questions: what shapes public and professional views of emerging genetic and biomedical technologies; who should bear responsibility for governing these technologies over time; and what values and governance frameworks should guide ELSI as it becomes more global, comparative, and institutionally embedded?

## 2 Publics, professionals, and the specificity of attitudes

A large cluster of papers in this issue examines the public and professional attitudes toward genomic and biomedical technologies. The papers point to a practical constraint on ELSI scholarship: attitudes toward genomics are not generic. They depend on the technology at issue, the setting in which it is encountered, the knowledge people bring, the worldviews they hold, and the risks and benefits they perceive (Greenbaum, 2025a). Publics do not encounter genomics in the abstract. They encounter genetic testing, genome editing, stem cell research, mRNA vaccines, brain organoids, microbiome engineering, and AI-enabled health systems, each embedded in particular institutional and moral settings.

DuBois et al. survey nearly 5000 U.S. adults on how religious factors shape support for genomic medicine across a range of activities, including postnatal genetic testing, storage and sharing of biospecimens and health data, genome editing, stem cell therapy and research, prenatal genetic testing, and mRNA vaccines (DuBois et al.). The central finding challenges dominant explanations in science communication: more strongly than political affiliation, education, or generalized institutional trust, specific belief-level and moral-worldview variables, particularly acceptance of evolution and moral commitments to health promotion within spiritual communities, emerge as the strongest predictors of support. Crucially, it is not religious affiliation *per se* that drives attitudes; the study finds only small differences between religious groups but substantial attitudinal variation within them. For ELSI scholarship and public engagement, religious identity is a poor proxy for moral reasoning: ELSI must attend not only to who people are, but to the interpretive worlds through which they understand genomic medicine.

Melchior et al. complement this picture from a methodological angle (Melchior et al.). By validating the Genetic Technologies Questionnaire for Greek-speaking populations, they provide more than a translated instrument: they show what comparative ELSI increasingly requires. Their findings reinforce the specificity of attitudes, with respondents distinguishing between conventional and genetic technologies, genetic testing and genome editing, and embryo and adult applications. These gradations matter because they show that publics do not hold a single attitude toward genetics. Rather, they make differentiated moral judgments across technologies, applications, and contexts. As ELSI becomes more comparative, it will need tools like the Genetic Technologies Questionnaire (GTQ): instruments that can travel across languages and settings without collapsing public attitudes into a generic view of genetic technology. Comparative ELSI cannot simply translate concepts across languages; it must test whether ethical categories travel well across social worlds.

Lee et al. extend the analysis to artificial intelligence in healthcare, surveying over 1,000 South Korean adults on their awareness and perceptions of AI ethics in health-related contexts, with direct relevance to genomic and health-data governance (Lee et al.). Their findings reveal a pattern that recurs across several papers in this issue: high optimism about technological potential coexists with significant concern about privacy, error, legal ambiguity, and the need for ethics education. A large majority of respondents expressed optimism about the positive impacts of AI in healthcare, yet concerns about personal information disclosure, AI-caused harm, and unclear legal responsibility were substantial. The study demonstrates that ELSI must increasingly engage with AI governance as a domain inseparable from genomic and health-data-driven medicine: as clinical decision-making passes through computational systems whose accountability structures remain underdeveloped, accountability must travel with the data.

A Japanese study further develops the public attitudes picture. Sawai, Kataoka, and Koike surveyed Japanese adults on brain organoid research and found broad support alongside concerns about unanticipated risks, commercialization, and personal information leakage (Sawai et al.). Notably, some concerns (about consciousness or cloning) may reflect misunderstandings, and comprehension scores were positively correlated with support. This suggests that science communication and governance must develop together: informed publics may view emerging research more favorably, but that does not remove the need for oversight.

Cummings examines U.S. public attitudes toward natural and genetically engineered microbiomes in the built environment, finding that topic-specific perceptions, particularly perceived benefits, risks, and quality-of-life improvements, predict support more strongly than demographic variables (Cummings). Their work exemplifies a responsible research and innovation (RRI)‐oriented empirical approach in which public perspectives are treated not as obstacles to innovation but as resources for shaping responsible development. It also expands the scope of ELSI beyond clinical and reproductive genomics to the engineered environments in which people live, work, and breathe. Public engagement, the study suggests, should accompany design choices from the outset rather than follow deployment.

Soares et al. shift the lens from public attitudes to professional uncertainty (Soares et al.). Their study of Portuguese clinical geneticists managing low-penetrance copy number variants reveals significant disagreement about disclosure practices, counseling strategies, and the ethics of prenatal diagnosis and preimplantation genetic testing for such variants, whose uncertain penetrance and variable expressivity create clinical and ethical challenges. The resulting variability in care, what the authors describe as a “postcode lottery” in which clinical outcomes depend on where a patient is seen rather than on the evidence itself, is a failure of distributive justice within a single national system. It also illustrates a point that public-attitude surveys alone cannot capture: genomic uncertainty is not only a challenge for patients deciding what to do with ambiguous information; it is equally a challenge for professionals who lack consensus about what to recommend.

Finally, Shaheen and Ghaly examine geneticization in the Gulf Cooperation Council region, attending to premarital screening, consanguinity, Islamic moral frameworks, family-centered decision-making, stigma, and the interaction of state modernization with genomic governance (Shaheen and Ghaly). This paper is one of the clearest demonstrations in the issue of why global ELSI cannot simply export Western secular-liberal assumptions into other contexts. In the GCC, genomic technologies are interpreted through distinctive religious, familial, legal, and public-health frameworks that shape their meaning in ways standard survey instruments alone cannot fully capture. Shaheen and Ghaly’s analysis broadens the geographic and cultural imagination of ELSI scholarship and makes a strong case for contextually grounded analysis.

More broadly, their paper invites us to view religious legal traditions not merely as sources of resistance or constraint, but as sophisticated normative systems capable of enriching public reasoning about technological authority, human agency, and institutional responsibility.

Taken together, these papers show that ELSI needs better comparative tools (shared instruments, coordinated multi-country studies, and theoretical frameworks that travel) but not at the expense of cultural and institutional specificity. The goal is not to flatten local moral worlds into a single global metric, but to build methods that allow meaningful comparison while preserving context.

## 3 Responsibility over time: from consent moments to governance relationships

A second cluster of papers addresses a temporal problem that runs throughout contemporary genomics: responsibility does not end when a genetic test is performed, a consent form is signed, or a policy is adopted. Knowledge changes. Variant classifications are updated. Databases grow. Regulatory environments shift. ELSI governance must therefore develop institutional models for ongoing obligation, not merely one-time authorization.

Onstwedder et al. use their 3-I framework (interests, ideas, and institutions) to examine policy considerations around incidental findings in large-scale genomic research (Onstwedder et al.). Their interview study with Canadian and European stakeholders shows that disagreements over incidental findings are not simply disagreements over ethical principles. They are also disagreements over institutional roles, stakeholder agendas, policy structures, and the distribution of benefits and burdens. Different jurisdictions allocate responsibilities differently, not because they hold different values in the abstract, but because their institutional architectures embed those values in different ways. The 3-I framework is valuable precisely because it makes these structural differences visible rather than treating policy divergence as a problem to be solved by better principle-articulation alone.

Sentell and Zawati address one of the clearest temporal challenges in genomic medicine: variant reclassification (Sentell and Zawati). As genomic knowledge evolves, variants previously classified as benign may be reclassified as pathogenic, or *vice versa*. But the duties to monitor emerging evidence, initiate reinterpretation, communicate updated results, and recontact patients remain ethically and legally underdefined. Their perspective proposes a shared-responsibility framework that distributes these obligations across laboratories, clinicians, patients, and health systems. This is a paradigmatic example of what the editorial identifies as the shift from consent-based to governance-based ELSI: the question is no longer only whether individuals consented at the beginning of a clinical encounter, but who remains responsible as interpretations change and new clinical actions become possible. The professional variability documented by Soares et al. is another expression of this governance problem: uncertainty in genomic interpretation becomes ethically consequential when institutions lack shared standards for disclosure, counseling, and reproductive decision-making (Soares et al.).

Peng et al. make the governance shift explicit in the domain of biospecimen data (Peng et al.). Their paper argues that traditional ownership models are inadequate for governing human biomedical data in the Chinese context and proposes custodianship, potentially implemented through data trusts, as an alternative. The urgency of this argument was underscored in 2025 when 23andMe, the direct-to-consumer genetic testing company, filed for bankruptcy, potentially exposing the genetic data of millions of customers and revealing the fragility of governance frameworks that rely on corporate privacy policies rather than durable institutional protections. The episode illustrates a broader structural shift: just as the commercialization of space required new regulatory frameworks when smaller enterprises entered a domain previously governed by and for national agencies, the proliferation of smaller genomic companies capable of assembling vast datasets demands governance architectures that were never designed with such actors in mind (Greenbaum and Gerstein, 2024). The conceptual move from ownership to custodianship reflects a broader trend visible across this collection: from individual control as the sole governance paradigm toward stewardship, relational responsibility, and institutional obligation. Their analysis also demonstrates that governance models must be adapted to particular legal, cultural, and political contexts; custodianship takes on different meanings and institutional forms in China, the European Union, and the United States.

## 4 Sovereignty, security, and the geopolitics of genomic governance

Genomic governance is increasingly entangled with questions of national sovereignty, biosecurity, cyberbiosecurity, industrial policy, and cross-border collaboration that extend well beyond the traditional ELSI agenda.

Song et al. provide a rare empirical window into this entanglement (Song et al.). Their analysis of Chinese administrative licensing data for human genetic resource governance between 2021 and 2024 shows what they describe as a regulatory pendulum: after the adoption of the 2023 Implementation Rules, the overall number of permits declined sharply, while the proportion of revocations increased. Yet international collaboration licenses continued to be issued, with U.S.-headquartered multinational corporations remaining the primary foreign partners. The picture that emerges is not one of simple closure or opening, but of differentiated, risk-calibrated, and sovereignty-conscious governance. China treats human genetic resources as strategic national assets, and its regulatory approach reflects the intersection of biosecurity, industrial strategy, scientific ambition, and geopolitical positioning.

Paired with Peng et al., these two papers make China a central site of analysis for understanding how genomic governance works in practice (Peng et al.; Song et al.). Song provides the administrative and empirical picture; Peng provides the normative argument for custodianship and responsible data sharing. Together, they complicate narratives that treat data governance as a binary choice between openness and restriction. Shaheen and Ghaly’s analysis of the GCC further reinforces this point: in that region too, genomic governance is shaped by regional identity, religious authority, national modernization projects, and public health goals in ways that cannot be reduced to a single regulatory model (Shaheen and Ghaly). The proposed move from ownership to custodianship reframes the central question from “who possesses the data?” to “who is entrusted to protect, steward, and responsibly share it?” (Greenbaum, 2021).

Global ELSI can no longer treat governance as a purely domestic matter. Human genetic resources, genomic data, clinical trials, and biotechnological infrastructures now sit at the intersection of ethics, law, diplomacy, security, and markets. The field needs analytical frameworks adequate to that complexity—and, as the next cluster of papers argues, governance institutions adequate to a more fundamental shift in what genomics is becoming.

## 5 From reading to writing: embedded ELSI and the governance of genomic design

If the preceding papers address who governs genomic knowledge and under what authority, two contributions in this issue ask a prior question: what kind of knowledge is genomics becoming, and what does that mean for how ELSI itself must be practiced?

Mikami’s perspective article provides the fullest conceptual articulation of this transition (Mikami). Mikami argues that ELSI was organized around what he calls the ELSI of eradication and the ELSI of knowing: questions about genetic disease, eugenics, prenatal testing, genetic counseling, incidental findings, and the consequences of possessing genomic knowledge. Genomics, however, is now moving into a “writing” paradigm centered on large-scale DNA synthesis, genome construction, and the redesign of living systems. This shift changes the ELSI agenda because the central question is no longer only what it means to know genetic information, but what it means to design, synthesize, and reconstruct organisms.

This design-oriented turn also resonates with earlier ELSI debates over human enhancement, where the difficulty of distinguishing therapy from enhancement revealed how regulatory and ethical judgments often depend less on the technology itself than on the purposes, contexts, and social meanings of its use (Greenbaum and Cabrera, 2020). Mikami therefore proposes an “ELSI of re-designing” that attends to scale, design choices, values embedded in technical decisions, and the distribution of power over what kinds of living systems are made. The shift from reading to writing genomes also intensifies questions of creator responsibility: what obligations do researchers, institutions, and regulators retain toward designed organisms and systems after they are made, and what are the consequences when those obligations go unfulfilled (Greenbaum, 2025b)? Mikami’s call for social scientists and humanities scholars to participate as collaborators rather than outside critics resonates with the broader emphasis across this issue on embedded, upstream engagement. Importantly, Mikami does not suggest that the ELSI of reading has become obsolete; genome writing adds a further layer of concern centered on design, scale, and participation, but the older questions about knowing, disclosing, and discriminating remain very much alive.

Matsuo et al. provide the institutional complement to Mikami’s conceptual argument (Matsuo et al.). Their policy and practice review calls for bottom-up ELSI and responsible research and innovation within Japan’s synthetic biology and biomanufacturing communities, identifying rule-making, proactive safety and security deliberation, and standardization as areas where researchers themselves should play a more active governance role. Rather than treating ELSI as an external review process or a top-down regulatory requirement, the paper argues for embedding anticipatory governance within research and development communities. That embedding carries its own risks: proximity to research communities can blunt the critical distance that makes ELSI scholarship valuable, and governance frameworks developed from within may be less equipped to challenge the assumptions on which a research program rests.

Together, Mikami and Matsuo suggest that ELSI is becoming less a downstream assessment of completed innovations and more an upstream and midstream practice integrated within research communities, funding structures, and regulatory institutions.

## 6 Values for the next stage of ELSI

Siermann et al. provide a useful conceptual anchor for synthesizing the collection as a whole (Siermann et al.). Their perspective, developed by a consortium of leading ELSI scholars, proposes three values as especially urgent for the changing landscape of genomics: equity, collective responsibility, and sustainability. Each runs through the papers in this issue.

Equity appears in Soares et al.'s documentation of the ‘postcode lottery’ in CNV counseling, in the uneven global distribution of genomic governance capacity, and in questions about who benefits from and who bears the burdens of incidental findings, AI-driven health systems, and cross-border data sharing (Lee et al.; Onstwedder et al.; Soares et al.). It also appears at a structural level: the capacity to conduct embedded, anticipatory, institutionally resourced ELSI is itself unevenly distributed, concentrated in high-income jurisdictions with established research infrastructures, while the populations most affected by genomic governance decisions frequently lack the institutional means to shape them.

Collective responsibility is visible in Sentell and Zawati’s shared-responsibility model for variant reinterpretation, in Peng et al.'s custodianship framework, in Matsuo et al.'s call for researcher-led governance, and in the broader shift across many papers from individual consent to institutional and relational obligation (Matsuo et al.; Peng et al.; Sentell and Zawati). Sustainability appears not only in environmental or resource terms, but as a question about the durability of governance systems: can laboratories, health systems, ethics committees, and regulators actually maintain the obligations that genomic medicine increasingly creates?

The older ELSI values (autonomy, privacy, informed consent, justice, non-discrimination) remain essential. But the papers in this collection suggest they are no longer sufficient on their own. Contemporary ELSI requires a thicker institutional vocabulary: custodianship, sovereignty, shared responsibility, and the ongoing stewardship of genomic knowledge and governance relationships over time. What is needed is not a replacement of the founding values, but an expansion adequate to a field that has moved from the sequencing laboratory to the clinic, the courtroom, the regulatory agency, the international negotiating table, and the public square.

## 7 Conclusion: from global expansion to comparative infrastructure

Across these contributions, the most important ELSI challenge is not simply ethical sensitivity but ethical scalability: ensuring that governance frameworks remain legitimate as genomic technologies move from exceptional interventions to routine infrastructure.

This Research Topic demonstrates how far ELSI has globalized and diversified. It is no longer U.S.‐centric, no longer focused exclusively on informed consent and privacy, and no longer purely theoretical. At the same time, the issue exposes a limitation that now matters for the field. Many of the papers still proceed as parallel national or regional conversations: the Japanese studies do not substantially engage the Korean or Chinese ones; the European governance papers and the GCC paper work from different intellectual starting points; and the survey studies use instruments that are not always easy to compare.

ELSI’s foundational vocabulary—consent, privacy, non-discrimination, the right not to know—reflected the era of reading genomes. The papers in this collection show a field that has moved well beyond those coordinates, toward the governance of genomic design, the geopolitics of data sovereignty, and the institutional architectures through which responsibility is distributed over time. Mikami’s call for an “ELSI of re-designing” captures the direction of travel (Mikami), and Lee et al.'s contribution points toward a further frontier: as AI systems increasingly mediate variant interpretation, literature synthesis, and clinical decision support, questions of opacity, data provenance, accountability, and error will become central to genomic governance (Lee et al.).

The next stage of ELSI will require more deliberate comparative infrastructure: shared instruments where comparison is useful, longitudinal designs where attitudes and responsibilities change over time, and cross‐national studies capable of identifying both convergence and divergence. But comparison must not come at the expense of context. Public attitudes, religious traditions, legal systems, clinical practices, and political institutions shape responsible genomics differently across settings. The contributors to this issue have advanced both projects. The task ahead is to bring them into closer dialogue, so that ELSI remains empirically grounded, normatively serious, and practically useful, measured not only by how well it critiques genomic innovation, but by how well it helps build the institutions through which genomic futures can be governed responsibly.
